# Supplementary material for: Modeling glioblastoma heterogeneity as a dynamic network of cell states
Source: Mol Syst Biol. 2021 Sep 16;17(9):e10105. doi: 10.15252/msb.202010105 (PMC8444284; doi:10.15252/msb.202010105)
Supplement: Supplementary file 6 — Source Data for Figure 5 [file MSB-17-e10105-s004.zip › Figure5A_sourcedata/GSEA_3071/nosigdb_stateI.GseaPreranked.1622795145664/RICHARDS_VERHAAK_CANCERCELL_2010_MESENCHYMAL.html]

Details for gene set RICHARDS\_VERHAAK\_CANCERCELL\_2010\_MESENCHYMAL[GSEA]

|  || Dataset | state13071 |
| Phenotype | NoPhenotypeAvailable |
| Upregulated in class | na\_pos |
| GeneSet | RICHARDS\_VERHAAK\_CANCERCELL\_2010\_MESENCHYMAL |
| Enrichment Score (ES) | 0.6160362 |
| Normalized Enrichment Score (NES) | 3.407523 |
| Nominal p-value | 0.0 |
| FDR q-value | 0.0 |
| FWER p-Value | 0.0 |
Table: GSEA Results Summary

  

Fig 1: Enrichment plot: RICHARDS\_VERHAAK\_CANCERCELL\_2010\_MESENCHYMAL      
 Profile of the Running ES Score & Positions of GeneSet Members on the Rank Ordered List

  

| PROBE | GENE SYMBOL | GENE\_TITLE | RANK IN GENE LIST | RANK METRIC SCORE | RUNNING ES | CORE ENRICHMENT || 1 | COL1A1 |  |  | 6 | 2.008 | 0.0399 | Yes |
| 2 | COL5A1 |  |  | 14 | 1.706 | 0.0733 | Yes |
| 3 | TGFBI |  |  | 17 | 1.674 | 0.1075 | Yes |
| 4 | S100A4 |  |  | 30 | 1.506 | 0.1353 | Yes |
| 5 | TIMP1 |  |  | 47 | 1.381 | 0.1593 | Yes |
| 6 | NRP1 |  |  | 81 | 1.180 | 0.1742 | Yes |
| 7 | P4HA2 |  |  | 82 | 1.171 | 0.1985 | Yes |
| 8 | SRPX2 |  |  | 90 | 1.123 | 0.2198 | Yes |
| 9 | CLEC2B |  |  | 95 | 1.105 | 0.2415 | Yes |
| 10 | CTSB |  |  | 129 | 0.964 | 0.2520 | Yes |
| 11 | NPC2 |  |  | 139 | 0.943 | 0.2689 | Yes |
| 12 | TNFAIP8 |  |  | 179 | 0.858 | 0.2754 | Yes |
| 13 | ANXA4 |  |  | 190 | 0.847 | 0.2901 | Yes |
| 14 | COL8A2 |  |  | 192 | 0.842 | 0.3073 | Yes |
| 15 | CEBPB |  |  | 222 | 0.782 | 0.3151 | Yes |
| 16 | SEC24D |  |  | 235 | 0.761 | 0.3274 | Yes |
| 17 | SCPEP1 |  |  | 259 | 0.727 | 0.3358 | Yes |
| 18 | EMP3 |  |  | 260 | 0.726 | 0.3508 | Yes |
| 19 | S100A11 |  |  | 275 | 0.707 | 0.3614 | Yes |
| 20 | DAB2 |  |  | 277 | 0.702 | 0.3757 | Yes |
| 21 | RUNX2 |  |  | 283 | 0.698 | 0.3888 | Yes |
| 22 | RBMS1 |  |  | 285 | 0.697 | 0.4029 | Yes |
| 23 | CAST |  |  | 298 | 0.675 | 0.4135 | Yes |
| 24 | S100A13 |  |  | 306 | 0.665 | 0.4252 | Yes |
| 25 | MYOF |  |  | 310 | 0.663 | 0.4381 | Yes |
| 26 | DSE |  |  | 312 | 0.659 | 0.4515 | Yes |
| 27 | GRN |  |  | 321 | 0.655 | 0.4628 | Yes |
| 28 | TRIM22 |  |  | 348 | 0.630 | 0.4683 | Yes |
| 29 | PROCR |  |  | 355 | 0.627 | 0.4796 | Yes |
| 30 | SHC1 |  |  | 361 | 0.624 | 0.4911 | Yes |
| 31 | TRIM38 |  |  | 386 | 0.602 | 0.4966 | Yes |
| 32 | LGALS3 |  |  | 390 | 0.600 | 0.5082 | Yes |
| 33 | LAMB1 |  |  | 415 | 0.577 | 0.5132 | Yes |
| 34 | LY96 |  |  | 451 | 0.547 | 0.5144 | Yes |
| 35 | SP100 |  |  | 466 | 0.538 | 0.5215 | Yes |
| 36 | CPQ |  |  | 488 | 0.523 | 0.5262 | Yes |
| 37 | PLBD1 |  |  | 500 | 0.511 | 0.5336 | Yes |
| 38 | ARHGAP29 |  |  | 508 | 0.502 | 0.5420 | Yes |
| 39 | IQGAP1 |  |  | 521 | 0.494 | 0.5488 | Yes |
| 40 | BLVRB |  |  | 535 | 0.488 | 0.5552 | Yes |
| 41 | SLC16A3 |  |  | 570 | 0.470 | 0.5550 | Yes |
| 42 | CASP4 |  |  | 585 | 0.460 | 0.5605 | Yes |
| 43 | PLS3 |  |  | 612 | 0.446 | 0.5622 | Yes |
| 44 | THBS1 |  |  | 613 | 0.446 | 0.5714 | Yes |
| 45 | MAN1A1 |  |  | 616 | 0.445 | 0.5801 | Yes |
| 46 | COPZ2 |  |  | 650 | 0.432 | 0.5795 | Yes |
| 47 | YAP1 |  |  | 657 | 0.430 | 0.5867 | Yes |
| 48 | FURIN |  |  | 663 | 0.426 | 0.5941 | Yes |
| 49 | CYBRD1 |  |  | 672 | 0.422 | 0.6005 | Yes |
| 50 | VDR |  |  | 681 | 0.420 | 0.6069 | Yes |
| 51 | MYH9 |  |  | 701 | 0.411 | 0.6099 | Yes |
| 52 | ARPC1B |  |  | 743 | 0.400 | 0.6063 | Yes |
| 53 | ADAM12 |  |  | 776 | 0.390 | 0.6051 | Yes |
| 54 | POLD4 |  |  | 810 | 0.378 | 0.6033 | Yes |
| 55 | LRRFIP1 |  |  | 824 | 0.374 | 0.6073 | Yes |
| 56 | MRC2 |  |  | 863 | 0.361 | 0.6037 | Yes |
| 57 | BNC2 |  |  | 864 | 0.361 | 0.6112 | Yes |
| 58 | ITGA5 |  |  | 874 | 0.357 | 0.6160 | Yes |
| 59 | CASP1 |  |  | 981 | 0.331 | 0.5921 | No |
| 60 | IGFBP6 |  |  | 1048 | 0.318 | 0.5795 | No |
| 61 | MGST2 |  |  | 1097 | 0.307 | 0.5719 | No |
| 62 | FXYD5 |  |  | 1117 | 0.303 | 0.5727 | No |
| 63 | RRAS |  |  | 1160 | 0.297 | 0.5666 | No |
| 64 | TMBIM1 |  |  | 1339 | 0.261 | 0.5203 | No |
| 65 | FHOD1 |  |  | 1369 | 0.257 | 0.5172 | No |
| 66 | TGFBR2 |  |  | 1410 | 0.251 | 0.5107 | No |
| 67 | SYNGR2 |  |  | 1939 | -0.312 | 0.3636 | No |
| 68 | CTSC |  |  | 1975 | -0.315 | 0.3600 | No |
| 69 | PYGL |  |  | 2071 | -0.329 | 0.3392 | No |
| 70 | DCBLD2 |  |  | 2407 | -0.393 | 0.2499 | No |
| 71 | UCP2 |  |  | 2498 | -0.413 | 0.2323 | No |
| 72 | HEXA |  |  | 2894 | -0.537 | 0.1286 | No |
| 73 | SERPINE1 |  |  | 3050 | -0.634 | 0.0967 | No |
| 74 | PDPN |  |  | 3472 | -1.788 | 0.0113 | No |
Table: GSEA details [plain text format]

  

Fig 2: RICHARDS\_VERHAAK\_CANCERCELL\_2010\_MESENCHYMAL: Random ES distribution      
 Gene set null distribution of ES for **RICHARDS\_VERHAAK\_CANCERCELL\_2010\_MESENCHYMAL**
